# Replication and Fine Mapping for Association of the *C2orf43*, *FOXP4*, *GPRC6A* and *RFX6* Genes with Prostate Cancer in the Chinese Population

**DOI:** 10.1371/journal.pone.0037866

**Published:** 2012-05-25

**Authors:** Qing-Zhi Long, Yue-Feng Du, Xiao-Ying Ding, Xiang Li, Wen-Bin Song, Yong Yang, Peng Zhang, Jian-Ping Zhou, Xiao-Gang Liu

**Affiliations:** 1 Department of Urology, the First Affiliated Hospital, School of Medicine, Xi’an Jiaotong University, Xi’an Shaanxi, People’s Republic of China; 2 Department of Anesthesia, the Second Affiliated Hospital, School of Medicine, Xi’an Jiaotong University, Xi’an Shaanxi, People’s Republic of China; 3 Department of Urology, The Affiliated Xi’an Ninth Hospital, School of Medicine, Xi’an Jiaotong University, Xi’an Shaanxi, People’s Republic of China; 4 Department of Urology, The Second Affiliated Hospital, School of Medicine, Xi’an Jiaotong University, Xi’an Shaanxi, People’s Republic of China; 5 Department of Urology, The Third Affiliated Hospital, School of Medicine, Xi’an Jiaotong University, Xi’an Shaanxi, People’s Republic of China; 6 The Key Laboratory of Biomedical Information Engineering of Ministry of Education, School of Life Science and Technology, Xi’an Jiaotong University, Xi’an Shaanxi, People’s Republic of China; University of Central Florida, United States of America

## Abstract

**Background:**

Prostate cancer represents the leading cause of male death across the world. A recent genome-wide association study (GWAS) identified five novel susceptibility loci for prostate cancer in the Japanese population. This study is to replicate and fine map the potential association of these five loci with prostate cancer in the Chinese Han population.

**Methods:**

In Phase I of the study, we tested the five single nucleotide polymorphisms (SNPs) which showed the strongest association evidence in the original GWAS in Japanese. The study sample consists of 1,169 Chinese Hans, comprising 483 patients and 686 healthy controls. Then in phase II, flanking SNPs of the successfully replicated SNPs in Phase I were genotyped and tested for association with prostate cancer to fine map those significant association signals.

**Results:**

We successfully replicated the association of *rs13385191* (located in the *C2orf43* gene, *P* = 8.60×10^−5^), *rs12653946* (*P* = 1.33×10^−6^), *rs1983891* (*FOXP4*, *P* = 6.22×10^−5^), and *rs339331* (*GPRC6A/RFX6*, *P* = 1.42×10^−5^) with prostate cancer. The most significant odds ratio (OR) was recorded as 1.41 (95% confidence interval 1.18–1.68) for *rs12653946*. *Rs9600079* did not show significant association (*P* = 8.07×10^−2^) with prostate cancer in this study. The Phase II study refined these association signals, and identified several SNPs showing more significant association with prostate cancer than the very SNPs tested in Phase I.

**Conclusions:**

Our results provide further support for association of the *C2orf43*, *FOXP4*, *GPRC6A* and *RFX6* genes with prostate cancer in Eastern Asian populations. This study also characterized the novel loci reported in the original GWAS with more details. Further work is still required to determine the functional variations and finally clarify the underlying biological mechanisms.

## Introduction

Prostate cancer (MIM 176807) represents the leading cause of male death in Western countries. With lifestyle transition, it has also become more and more prevalent in Asia. It is widely accepted that genetics play important roles in susceptibility to almost all human cancers. Now, genome-wide association study (GWAS) has become a feasible, powerful and effective approach for identification of novel genes underlying susceptibility to cancers and other complex diseases.

A recently conducted GWAS on prostate cancer identified five novel susceptibility loci in the Japanese population [1]. These loci had not been associated with prostate cancer before. Investigation in diverse populations of the variants discovered from GWASs may contribute to a more comprehensive understanding of genetic mechanisms of prostate cancer. Here, we report our replication efforts in the Chinese Han population for potential association of these five loci with prostate cancer. Specifically, five SNPs that showed the strongest association evidence respectively in their situated loci in the original GWAS were genotyped and tested for potential association with prostate cancer. This is the first replication study on these five novel loci with prostate cancer in the Chinese population. Furthermore, as an effort to fine map the replicated association signals, we genotyped a total of another 259 SNPs encompassing the successfully replicated SNPs and tested for their association with prostate cancer.

## Materials and Methods

### Ethics Statement

The study was approved by the institutional review board of Xi’an Jiaotong University. Signed informed-consent documents were obtained from all study participants.

### Study Sample

The study sample consisted of 483 patients and 686 healthy geography- and age-matched controls. The patients were recruited from in- and outpatients of the participating hospitals from 2007 to the present. All patients were independently and consistently diagnosed by two senior urologists and a senior pathologist based on medical records and pathological evaluation of prostate biopsy. We preferentially recruited patients who had a self-reported family history of prostate cancer or a high-grade Gleason score (≥7) at the time of diagnosis. Each study subject filled in a structured questionnaire form covering family history, medical history, alcohol consumption, smoking, diet structure, socioeconomic factors, etc. The control subjects were drawn from a growing database comprising more than 3,000 randomly-enrolled unrelated Han Chinese from Xi’an city and its neighboring area. Individuals having serious chronic diseases/conditions that may have potential influence on endocrine and metabolism were excluded. The inclusion and exclusion criteria have been described in details elsewhere [2], [3]. Potential confounding factors, including native place, alcohol consumption, smoking, diet structure and socioeconomic factors, were maximally considered to match the patient and control groups.

### SNP Selection and Genotyping

In Phase I, we selected the five SNPs that showed the strongest association evidence respectively in their situated loci in the original GWAS. As for Phase II, the SNP markers were selected based on a comprehensive consideration of: (1) coverage of the coding sequence and up to 300 kbp upstream of the gene under scrutiny; (2) validation of the SNP from both dbSNP and HapMap databases; (3) degree of heterozygosity, i.e., minor allele frequency ≥5%; (4) inclusion of the SNP in Affymetrix and Illumina SNP chips. This is for convenience of comparison of association results between this study and others.

Genomic DNA was extracted from peripheral blood leukocytes applying standard protocols. SNP genotyping was performed with a primer-extension method with MALDI-TOF mass spectrometry on a MassARRAY system as suggested by the manufacturer (Sequenom, Inc., San Diego, CA). Genotype frequencies of all the five SNPs in both case and control groups did not deviate significantly from Hardy-Weinberg equilibrium (*P*>0.05). In phase II, 66, 40 and 81 SNPs were successfully genotyped and past the Hardy-Weinberg equilibrium test, respectively for the loci around the *C2orf43* (Chromosome 2 Open Reading Frame [MIM 613570]), *FOXP4* (Forkhead Box P4 [MIM 608924]) and *GPRC6A/RFX6* (G Protein-coupled Receptor, family C, group 6, member A [MIM 613572] and regulatory factor X, 6 [MIM 612659]) genes. Given *rs12653946* was not mapped to any known gene, 72 flanking SNPs of *rs12653946* were genotyped in phase II.

### Statistical Analyses

The genotype distributions of the tested SNPs between case and control groups were analyzed with logistic regression models controlling for age and alcohol consumption as covariates. In the frame of logistic regression model, odds ratio (OR) with the corresponding 95% confidence intervals were also computed. We adopted the conservative Bonferroni method to account for the multiple-testing problem respectively for the study of Phase I and Phase II. Because five SNPs were tested in the replication phase, the significance level was set as 1.00×10^−2^ (i.e., 0.05/5). In phase II, the significance level for the study was set as 1.93×10^−4^ (i.e., 0.05/259).

We utilized online FASTSNP (http://fastsnp.ibms.sinica.edu.tw) in order to analyze the potential functions of the pinpointed SNPs. The analysis is based on up-to-date information extracted from 11 external bioinformatic databases at query time [4].

## Results

Clinical characteristics of the patient subjects are presented in Table 1. The average age of patients and controls was 67.3±6.9 and 66.3±6.4, respectively. All study subjects are Han Chinese recruited from the Xi’an city and its neighboring area. Our previous studies [2], [3] did not detect significant population stratification in the study sample database, from which all the control subjects of this study were drawn. Some interesting characteristics of the case and control samples are presented in Table 2.

**Table 1:** Clinical characteristics of the patient subjects.

	Number (%)	
Tumor stage		
T0	4	(0.8)
T1	47	(9.7)
T2	70	(14.5)
T3	41	(8.5)
T4	9	(1.9)
Missing data	310	(64.2)
Nodal stage		
N0	144	(29.8)
N1	11	(2.3)
Missing data	327	(67.7)
Metastasis stage		
M0	179	(37.1)
M1	13	(2.7)
Missing data	290	(60.0)
Gleason score		
GS <7	120	(24.8)
GS ≥7	143	(29.6)
Missing data	219	(45.3)

**Table 2:** Characteristics of the case and control samples.

	Casen = 483	Controln = 686
Average Age (years, ± SD)	67.3 (±6.9)	66.3 (±6.4)
Average Height (m, ± SD)	161.4 (±8.6)	162.1 (±8.8)
Average Weight (kg, ± SD)	62.6 (±10.6)	63.2 (±10.2)
Percentage of regular smokers^a^	46.8%	45.1%
Percentage of regular alcoholconsumers^b^	31.2%	28.9%
Annual household income (RMB)	31,200	33,600

**Note:** Abbreviations: SD, standard deviation;

RMB, RenMinBi, the legal tender of mainland China;

a Regular smokers refer to subjects who smoke at least half a pack of cigarettes per day;

b Regular alcohol consumers refer to subjects who drink at least two times per week.

Our results of the Phase I study provided supportive evidence for four of the five tested SNPs as true susceptibility loci of prostate cancer. We confirmed association of *rs13385191*, *rs12653946*, *rs1983891*, and *rs339331* at *P* = 8.60×10^−5^, 1.33×10^−6^, 6.22×10^−5^, and 1.42×10^−5^, respectively, with the same high-risk alleles to those in the original GWAS in the Japanese population. Characteristics of the studied SNPs and corresponding association test results in the replication phase are showed in Table 3.

**Table 3:** Association test results and characteristics of the studied SNPs in the replication phase.

dbSNP ID	Association test results		OR^a^	Region^b^	Location^a^	Role	Gene^b^	Alleles^c^	Minor allele and MAF			
	Original GWAS	Our data							HapMap			Our data
									CHB	JPT	CEU	
*rs13385191*	7.5×10^−8^	8.60×10^−5^	1.33 (1.11–1.58)	2p24	20751746	Intron	*C2orf43*	G/A	G(0.49)	A(0.37)	G(0.20)	A(0.44)
*rs12653946*	3.9×10^−18^	1.33×10^−6^	1.41 (1.18–1.68)	5p15	1948829	/		T/C	T(0.35)	T(0.43)	T(0.43)	T(0.39)
*rs1983891*	7.6×10^−8^	6.22×10^−5^	1.34 (1.13–1.59)	6p21	41644405	Intron	*FOXP4*	T/C	T(0.37)	T(0.38)	T(0.27)	T(0.38)
*rs339331*	1.6×10^−12^	1.42×10^−5^	1.39 (1.16–1.65)	6q22	117316745	Intron	*GPRC6A/RFX6*	T/C	C(0.35)	C(0.40)	C(0.36)	C(0.37)
*rs9600079*	2.8×10^−9^	8.07×10^−2^	1.07 (0.88–1.27)	13q22	72626140	/		T/G	T(0.48)	T(0.32)	T(0.46)	T(0.39)

**Note:** MAF, minor allele frequency. UTR, untranslated region;

a Odds ratio followed by 95% confidence interval;

b Region and location are based on HapMap data release#24;

b Blank cells mean the SNPs are not mapped to any gene;

c The risk allele is placed prior to the slash.

**Figure 1:**
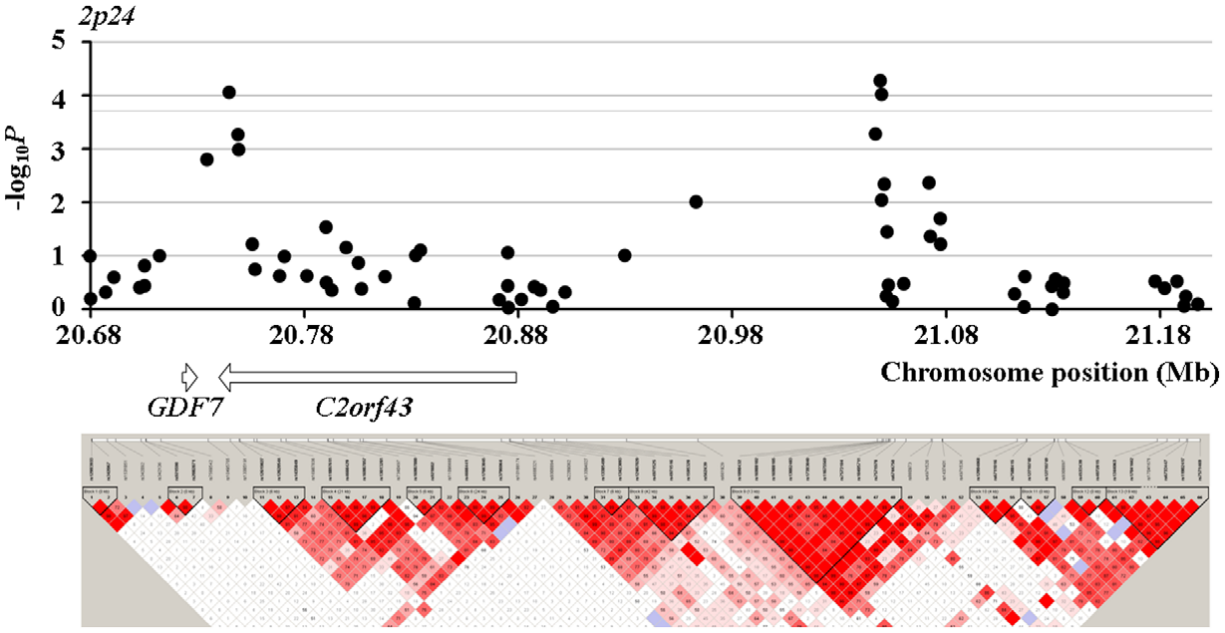
LD maps of the tested SNPs and association test results for the loci around *rs13385191* in Phase II.

**Figure 2:**
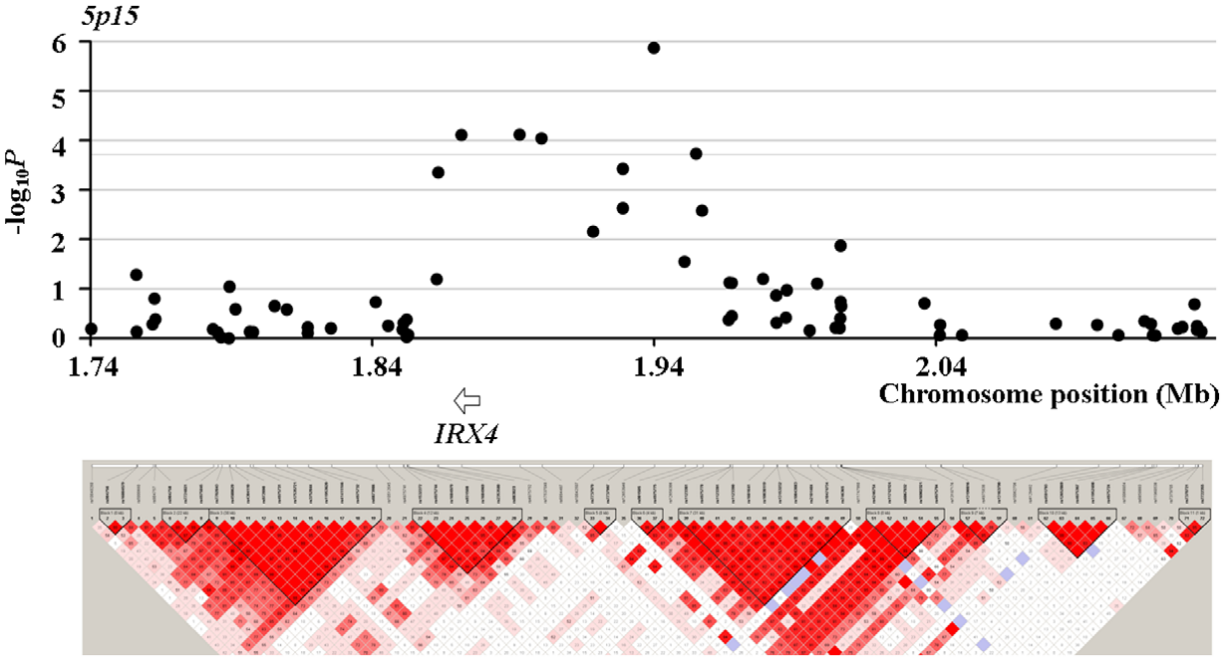
LD maps of the tested SNPs and association test results for the loci around *rs12653946* in Phase II.

**Figure 3:**
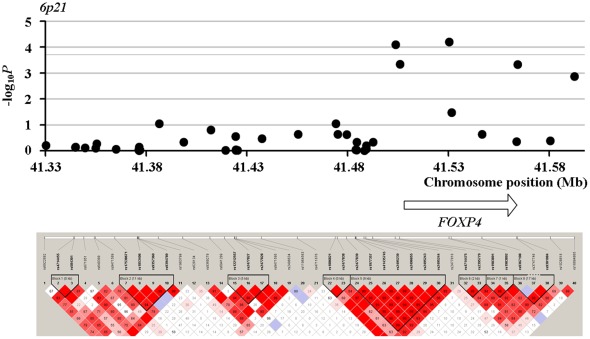
LD maps of the tested SNPs and association test results for the loci around *rs1983891* in Phase II.

**Figure 4:**
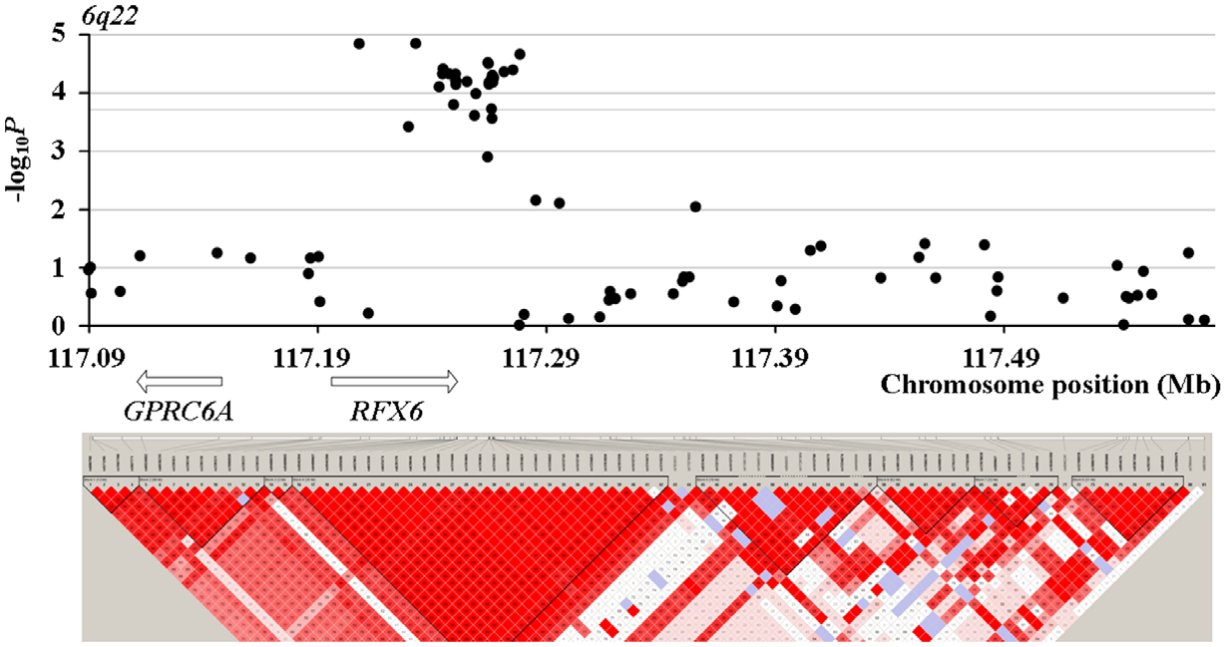
LD maps of the tested SNPs and association test results for the loci around *rs339331* in Phase II.

In Phase II, we found two SNPs showing even stronger association than their corresponding two SNPs tested in Phase I. *Rs16988102* obtained a *P* value of 5.29×10^−5^ compared to *rs13385191* with a *P* value of 8.60×10^−5^. *Rs9489065* got a *P* value of 1.40×10^−5^, compared to *rs339331* with a *P* value of 1.42×10^−5^. When jointly modeling prostate cancer with *rs16988102* and *rs13385191*, the OR (95% CI) increased to 1.71 (1.47–1.96), compared to OR of 1.33 (1.11–1.58) when considering *rs13385191* alone. When jointly modeling the four SNPs which got the most significant association signals in their respective loci, the OR (95% CI) turned out 2.06 (1.79–2.35). LD (linkage disequilibrium) structure of the tested SNPs and results of the association tests in Phase II for the four loci around *rs13385191*, *rs12653946*, *rs1983891*, and *rs339331* are presented in Figure 1, 2, 3 and 4. The LD map was generated by the use of Haploview (Broad Institute, MA, USA) based on D-Prime using the genotype data of case and controls in the Phase II study.

## Discussion

*Rs13385191* is located in intron region of the *C2orf43* gene, which encoding a hypothetical protein LOC60526. The *C2orf43* gene is conserved among chimpanzee, cow, mouse, rat, chicken, zebrafish, fruit fly, mosquito, C.elegans, A.thaliana, and rice. This suggests the existence of important functional variants around the gene region. Notably, the chromosome region 2p24 harboring the *C2orf43* gene was linked to prostate cancer in European and American populations [5]. This complies with the detected association of the *C2orf4*3 gene with prostate cancer in this study. FASTSNP analyses suggested that *rs13385191* may be a location of intronic enhancer.

*Rs1983891* is mapped to intron region of the *FOXP4* gene. The *FOXP4* gene was first reported as a novel forkhead transcription factor [6]. FOXP4 belongs to subfamily P of the forkhead box (FOX) transcription factor family. Forkhead box transcription factors play important roles in the regulation of tissue- and cell type-specific gene transcription. Many members of the forkhead box gene family, including members of subfamily P, participate in mammalian oncogenesis [6]. The *FOXP4* gene is located at chromosome region 6p21, which region was also ever linked to prostate cancer [5], [7].

*Rs339331* resides close to two genes, *GPRC6A* and *RFX6*. FASTSNP showed that *rs339331* may be a location of intronic enhancer of the *GPRC6A* gene. Prostate does not express GPRC6A in normal conditions [8]. Nevertheless, interestingly, GPRC6A is highly expressed in the Leydig cells of the testis, and mice deficient in Gprc6a show male feminization and a metabolic manifestation of higher circulating estradiol and reduced levels of testosterone. These two hormones are critical for initiation and progression of prostate cancer [9]. As another supporting evidence for the significant association, GPRC6A is functionally important in regulating non-genomic effects of androgens in multiple tissues [10]. *Rs339331* resides in the chromosome region 6q22, which was found to be a susceptibility loci of prostate cancer in US Whites [11].

The *RFX6* gene encodes a member of the regulatory factor X (RFX) family of transcription factors [12]. The coded DNA-binding protein RFX6 plays an important role in the pathology of neonatal hemochromatosis [13]. Association of the *HFE* (hemochromatosis) gene with prostate cancer [14] highlight the crosstalk between pathology of hemochromatosis and prostate cancer.

*Rs12653946* and *rs9600079* are not mapped to any known gene. Nevertheless, 5p15, the locus harboring the *rs12653946* polymorphism, was ever linked to prostate cancer aggressiveness [15], [16]. This is in accordance with the detected association of *rs12653946* with prostate cancer in this study.

Possible reasons for failure of replication for *rs9600079* might include different ethnic backgrounds, difference in selection criteria of study subjects, and/or ethnic heterogeneity of genetics mechanisms of prostate cancer.

In Phase II of fine mapping for the replicated loci in Phase I, *rs16988102* and *rs9489065* got more significant association signals with prostate cancer than their neighboring SNPs replicated in Phase I. The association of *Rs16988102* with prostate cancer was also supported by strong associations of multiple surrounding SNPs with prostate cancer. *Rs16988102* is located in 5′ upstream of the *C2orf43* gene. The distance between *rs16988102* and *rs13385191* is more than 300 kb, which is much longer than general distance of LD. Thus, we prefer to claim the *rs16988102* as a risk locus for prostate cancer independent to *rs13385191*. This is in accordance with the significantly lifted OR by adding *rs16988102* to the risk model of prostate cancer which only took the variation of *rs13385191* into account. *Rs9489065* is located in intron region of the *RFX6* gene. *Rs9489065* and *rs339331* are in strong LD (D′ = 0.97), and their association signals with prostate cancer are largely comparative. This implies that one of *rs9489065* and *rs339331* is the true susceptibility loci, or that both these two SNPs are in strong LD with the true susceptibility loci. Notably, around the loci of the *GPRC6A* and *RFX6* genes, the SNPs significantly associated with prostate cancer narrowed to the region of the *RFX6* gene, compared to a wide distribution of significantly associated SNPs across the whole region of these two genes in the original GWAS. This suggests the *RFX6* gene variation may be the susceptibility loci underlying the observed association signal of the *GPRC6A/RFX6* loci with prostate cancer in the original GWAS.

In the original GWAS, Takata *et al.* failed to replicate association of 12 SNPs among the total of tested 31 SNPs which are significantly associated with prostate cancer in European populations. This highlighted the genetic heterogeneity of prostate cancer among diverse ethnic populations. In this study, four among five tested SNPs which are significantly associated with prostate cancer in Japanese are successfully replicated in Chinese Han population. Compared with the replication ratio of 12/31, the sharply higher replication ratio of 4/5 raised the possibility of similar genetic pathology of prostate cancer between the Japanese and Chinese Han populations. This is not unexpected given the genetic homogeneity between the Chinese and Japanese populations [17].

In summary, our results provide further evidence for significant association of SNPs *rs13385191*, *rs12653946*, *rs1983891*, and *rs339331* with susceptibility to prostate cancer in Eastern Asian populations. The related genes, *C2orf43*, *FOXP4*, *GPRC6A* and *RFX6*, are warranted for further efforts to determine the functional variations and finally to clarify the genetic mechanism of susceptibility to prostate cancer.
